# Guidelines for Caring for Incapacitated Teachers and Students at Former Jesuit Colleges in Europe

**DOI:** 10.1007/s10943-024-02198-y

**Published:** 2024-12-05

**Authors:** Jerzy Kochanowicz, Beata Topij-Stempińska, Piret Paal

**Affiliations:** 1 Faculty of Applied Sciences, Department of Education, WSB University, Cieplaka 1C, 41-300 Dąbrowa Górnicza, Poland; 2 Faculty of Education, Institute of Educational Sciences, Ignatianum University in Cracow, Kraków, Poland; 3 Department of Ethnology, Institute of Cultural Studies, University of Tartu, Tartu, Estonia; 4 Institute of Palliative Care, Paracelsus Medical University, Salzburg, Austria

**Keywords:** Health, Illness, History of medicine, Education, Jesuit colleges

## Abstract

The aim of this article is to explore the regulations governing the treatment of incapacitated people in former Jesuit colleges in Europe, focusing on the academic staff and students residing in these institutions. This treatment was strongly influenced by the spirituality of Ignatius of Loyola, who perceived disease in dual terms: as an evil to be combated through all available means and as a test of faith from God. Ignatius instructed college superiors to prioritize the care of the sick and appointed specific officers within the community, such as prefects of health, nurses, and pharmacists, while also formulating detailed rules for the care and treatment of the sick. Understanding these principles, which served as a *vademecum* for nursing practices at the time, provides insights into the daily life of former Jesuit colleges and can serve as inspiration for modern approaches to patient care, particularly emphasizing the importance of attending to their mental well-being.

## Introduction

Cultural, philosophical, and religious beliefs significantly influence societal perspectives on health and illness. In Christianity, there is a strong emphasis on compassion and care for the sick demonstrated through practical aid, emotional support, and prayer. Despite acknowledging suffering as inherent to human existence, Christianity emphasizes the importance of supporting the sick through both practical assistance and spiritual solidarity (Koenig et al., 2024).

These principles are evident in the activities of the Catholic order of the Society of Jesus (SJ), founded in 1540 by Ignatius of Loyola (1491–1556). Originally focused on charitable works, such as assisting the sick and imprisoned, members of the Society of Jesus, popularly called Jesuits, later shifted their focus to education, becoming renowned for their schools and universities (O’Malley, 1995). Even within their educational institutions, they prioritized caring for incapacitated professors and students.

To comprehend this aspect of their mission, it is essential to examine the education provided by the order until 1773. The Society of Jesus managed hundreds of colleges offering secondary education, some of which functioned as universities. These institutions welcomed students from diverse backgrounds, although noble families increasingly predominated over time. Students were categorized into three groups: young Jesuits residing within the college, pursuing studies in philosophy or theology; residents of various boarding houses supervised by Jesuits, including theological seminaries, papal boarding schools, noblemen’s boarding schools, and poor people’s dormitories (later transformed into music seminaries); and a larger group of students living either at home or in boarding houses under parental care (Kochanowicz & Topij-Stempińska, 2020).

All students followed a standardized educational curriculum structured by their class or faculty. The teaching staff mainly comprised Jesuits, with exceptions such as singing instructors and lay professors of law and medicine. Jesuit teachers were divided into clerics, who taught lower grades, and priests, who instructed older grades or delivered lectures in philosophical and theological studies (Grendler, 2018).

The Jesuits, influenced by Renaissance educational principles (Erasmus, Vives, Rabelais, More, Elyot), emphasized a comprehensive approach to education, integrating moral and intellectual development with recreation and physical activity essential for effective learning (Padberg, 1996, no. 339). They prioritized the well-being of both faculty and students, believing that good health was crucial for intellectual pursuits. In cases of illness, every effort was made for recovery.

## Methods

This article aims to explore the regulations governing the treatment of impaired academic staff and students within former Jesuit colleges. It seeks to uncover the religious justifications behind this care, examine the institutional forms it took, and investigate the rules that applied to medical staff.

The study focuses on three groups of students: young Jesuits studying philosophy or theology, residents of various boarding houses supervised by Jesuits, and students living at home or in parental care boarding houses. The selection criteria for these groups are based on their direct involvement and residency within Jesuit educational institutions, ensuring a comprehensive understanding of the care practices applied to them.

By "former" Jesuit colleges, we refer to the educational institutions operated by the Society of Jesus from its foundation in 1540 until the order’s suppression in 1773. We limit our inquiry to the era during which Jesuit educational and healthcare practices were well-documented and widely implemented across their institutions.

Contemporary Jesuit colleges around the world refer in their educational ideology to the principles established at the beginning of the Jesuit Order. However, they are not required to follow the provisions of a single document (Ratio Studiorum) and must adapt their practices to the educational legal requirements of the countries in which they operate.

Despite extensive discussions on Ignatius of Loyola’s views on illness (e.g., Welie, 2003), these specific issues have received less attention in the literature. To address this gap, this study employs a historical method to investigate the approach to caring for sick individuals in former Jesuit colleges. We began by formulating a research question to steer our analysis and gathered a diverse array of primary sources, including writings by Ignatius (like his letters and the Constitutions of the Society of Jesus), alongside secondary sources, such as interpretations by other authors, all of which are relevant to our inquiry (e.g., Arzubialde, 2007; Dalmases, 1985; Mariani, 2021; Welie, 2003).

Specifically, we focused on Ignatius’s principles concerning illness and care, as well as directives given to college superiors. Additionally, we scrutinized guidelines from subsequent Jesuit leaders regarding care for the sick, comprehending the roles of key individuals such as the prefects of health, nurses, and pharmacists. Since the texts were already available in critical editions, we deemed source criticism unnecessary. We integrated these diverse sources to construct a comprehensive narrative and offer a detailed account of themes. By categorizing data according to common topics, we delved into underlying meanings and interpretations across different sources (Carr, 2001).

We adopted a historical perspective to understand the foundational principles and practices of care within former Jesuit educational institutions. By examining the period up to 1773, we aimed to gain insights into the origins and evolution of these practices, which were rooted in Jesuit philosophy and the broader socioreligious context of the time. This diachronic approach allowed us to trace the development of care practices and their long-term impacts, whereas a synchronic approach would only have provided a snapshot of contemporary practices.

## Illness as an Instrument of Divine Pedagogy according to Ignatius of Loyola

### The Role of Illness in Ignatius’s Spiritual Awakening

Ignatius of Loyola was perceived by his contemporaries as a man of immense mental strength and determination. However, few were aware that he struggled with serious health issues for years. In May 1521, while serving as a soldier in the Spanish garrison in Pamplona, he suffered a severe wound, leading to a lengthy and painful convalescence (Loyola, 1943, p. 366). Influenced by devout reading, his life underwent a profound transformation during this period: once a proud knight, he became a humble pilgrim, and eventually, the founder of the Jesuits. He envisioned this new order as one dedicated to “compassionately assist and serve those who are in prisons or hospitals, and indeed to perform any other works of charity” (Formulas of the Institute, 1996, p. 4).

### Indifference Toward Health and Sickness in Jesuit Spiritual Teachings

Regarding health and sickness, Jesuits should remain indifferent. In the “Principle and Foundation” of his *Spiritual Exercises*, Ignatius taught that one should prefer neither health over sickness nor wealth over poverty but instead focus solely on what aids in serving God and saving the soul. All created things, including health and sickness, should be valued only in terms of their contribution to this spiritual goal, reflecting a deep detachment from worldly concerns and a commitment to divine purpose (Loyola, 2017).

Ignatius perceived illness (both physical and mental) and the suffering associated with it primarily as one of life’s trials (*pruebas*) that God sends to man (Arzubialde, 2007). Hence, he held the belief that amidst illness, one should shift focus away from personal suffering and instead recognize it as a test from a benevolent God (referencing Hebrews 12:6, “the Lord disciplines the one he loves”). According to Ignatius, illness holds equal significance for health, as it could serve as a catalyst for spiritual awakening. However, this newfound fervor might lead to overzealousness in the early stages of conversion.

### Moderation: The Jesuit Approach to Health and Lifestyle

In the case of Ignatius, the effects of this conversion included his excessive penitential practices, which caused him chronic stomach problems that plagued him throughout his life. The pain and high fever sometimes lasted for several hours, forcing Ignatius to stay in bed (Loyola, 1943, p. 489). For this reason, he had to interrupt his theological studies at the University of Paris and was close to death three times. After founding the Jesuit Order, he categorically warned young Jesuits against strict penitential practices (Loyola, 1903b, pp. 499–503) and designed an order that, compared to other congregations, was characterized by extraordinary gentleness in the field of mortification, at least at the beginning of its existence.

Ignatius highly prioritized good health, believing it to be of utmost importance. Consequently, he advocated that Jesuits, including both teachers and students, should refrain from engaging in activities that could harm their health through excess. Instead, they should consistently adhere to moderation (*discretio*), which represents a manifestation of reasonable love (*caritas discreta*). This principle of moderation should be applied across various aspects of life, including work, study, rest, meals, religious practices, and any other activities that could impact one’s health (Rotsaert, 2024). Ignatius envisioned that moderation, derived from a thoughtful assessment of external circumstances and internal needs, would become a defining characteristic of both the Jesuit Order and the educational approach adopted within its institutions.

### The Transformative Experience of Serving the Sick

The attitudes of Ignatius and his first nine companions toward disease and sick people were significantly shaped by the events of 1537. During this time, they traveled from Paris to Venice with the intention of boarding a ship bound for the Holy Land. However, their journey was delayed due to the ongoing war between Venice and the Turkish Empire. Consequently, they found themselves staying in two local hospitals—SS. Giovanni e Paolo and the Ospedale degli Incurabili, which catered to the incurably ill (Loyola, 1903a).

After completing advanced studies and attaining mastery in philosophy at the renowned Sorbonne, they dedicated many weeks to serving the sick within these hospitals. Their tasks included menial and physically demanding chores such as washing patients, making beds, cleaning floors, offering spiritual support through confession, preparing individuals for death, and handling the deceased by carrying out bodies and digging graves. Ignatius described these duties as “most demeaning and physically repugnant” (Loyola, 1903a, p. 119).

This transformative experience in Venice had a lasting impact on the Jesuits, shaping their identity and becoming a foundational myth within the order. It solidified their commitment to caring for the sick, with the ethos emerging that a Jesuit is one who compassionately attends to those in need.

### Formation and Testing of Novices Through Hospital Service

When Ignatius later made recommendations regarding the formation of candidates for the order, he included, among the various tests (*pruebas*) they had to undergo, an obligation to serve in a hospital for a month. According to the Constitutions of the Society of Jesus, novices were required to “take their meals and sleep in it […]. They should help and serve all, the sick and the well, in conformity with the directions they receive, in order to lower and humble themselves more, thus giving clear proof of themselves to the effect that they are completely giving up the world with its pomps and vanities” (no. 66).

Given that most candidates for the order came from wealthier social classes, often from the nobility, and considering the unsanitary conditions of hospitals at that time, this obligation posed a significant challenge for young Jesuits. It served as a test of the authenticity of their religious motivation. Nonetheless, it also ingrained in them, right from the outset, the conviction that tending to the ill held not just less but indeed greater significance than the esteemed and glamorous duties they might later undertake in royal courts or academies. Taking care of the sick was intended to ground proud and well-educated Jesuits, reminding them of the humility and compassion central to their calling.

### Letters of Ignatius: Insights on Illness as Divine Pedagogy

An invaluable source for understanding Ignatius of Loyola’s perspectives on illness and the sick is his extensive collection of letters, which served as a source of inspiration and guidance for the Society of Jesus. Ignatius wrote nearly 7,000 letters on various topics throughout his life, 28 of which specifically addressed the issue of disease and health. In these letters, he portrays illness primarily as a tool of divine pedagogy (*paideia divina*).

Ignatius suggests that illness often, though not always, originates from the hand of God, who uses it to demonstrate his love for humanity. According to Ignatius, God’s primary concern is to heal the diseases of the human soul while also testing individuals’ patience and love.

In his letters, Ignatius delineated six objectives that God seeks to accomplish within individuals through illness as a component of his divine educational process (Arzubialde, 2007) (see Table 1).

**Table 1:** Ignatian’s six objectives of god for human accomplishment

1. Illness offers an opportunity for individuals to purify themselves of their sins here on Earth through the suffering it entails
2. The experience of illness provides a chance for self-reflection, leading to the attainment of the virtue of humility
3. By isolating man from his cherished possessions and activities, God aims to highlight the fragility and brevity of life, thereby lessening his attachment to worldly things and fostering greater freedom in loving God
4. Through illness, individuals can cultivate virtues such as patience and trust in God alone
5. God desires individuals to align their will with the supreme and eternal will of God the Father, mirroring the submission of Christ
6. Illness enables individuals to share in the joy of Christ’s suffering, reinforcing the bond between Christ and the sufferer

After delineating the divine pedagogy inherent in illness, Ignatius directs attention to men’s attitudes toward illness. According to the founder of the Jesuits, this attitude should be twofold. First, the patient is expected to utilize all available means to seek relief and facilitate recovery (Loyola, 1908). This endeavor for swift recuperation is deemed the primary duty of the sick individual, to the extent that prayers and devout readings may be curtailed if they impede recovery. Second, a sick Jesuit, as well as a student in a Jesuit college, should embrace all afflictions with patience, calmness, gratitude, and trust as expressions of God’s paternal love, whether they are perceived as disciplinary or nurturing (Arzubialde, 2007). Once again, this stance underscores the Jesuit perspective of intellectual engagement with reality: illness is acknowledged as a negative force to be combated vigorously while simultaneously seeking deeper meaning in afflictions.

### Ignatius of Loyola’s Vision: Aligning Life’s Purpose with Divine Guidance

Ignatius of Loyola firmly believed that the primary objective of every individual’s life is to glorify the Creator through virtuous living and impactful deeds. However, he recognized that achieving this goal requires inner harmony, nurtured by a trusting openness to divine guidance, even when it manifests through illness. The Jesuits inherited this perspective on illness from their founder and endeavored to impart it to their students as well.

This study explores four essential aspects of internal alignment:

- The rector’s duty to care for sick residents of the college
- Responsibilities of the health prefect
- Rules for the infirmarian: nursing guidelines
- The responsibilities of the pharmacist.

## The Rector’s Duty to Care for Sick Residents of the College: Following the Example of Ignatius

The Society of Jesus implemented stringent criteria for admitting individuals with sound health to its ranks. Despite meticulous selection upon entry into the order, numerous Jesuits fell victim to various illnesses, given the diverse array of activities they engaged in. Ignatius of Loyola grappled with the issue of ill Jesuits early on. Historical accounts from the inception of the Society of Jesus reveal that as the superior general and legislator of the order, he exerted every effort to ensure that all members maintained good health to serve God more effectively. However, if someone did become ill, they were to receive special care.

As Ignatius’s twenteeth-century biographer Candido de Dalmases notes, “One of the clearest proofs of his love for his subjects was the special and minute concern which he showed for the sick. Manifestations of it, among others, were these: his asking the house buyer to inform him twice each day if he had bought all that the infirmarian had requested; his imposing penances for incidents of negligence in attending to the sick; and his ordering the rector of a college to inform him when anyone fell sick. When the needs of the sick were concerned, he did not look closely at expenses. He once sold some pewter plates that the house possessed to be able to buy the prescribed medicines. He said that, if necessary, even the sacred vessels would have to be sold” (Dalmases, 1985, p. 258).

Dalmases continues: “He took care of the sick personally and served them with great humility and charity, as though he had nothing else to do. […] On one occasion, he delegated the functions of superior of the house to Nadal, but he reserved to himself what pertained to the care of the sick. […] To supply relaxation for the students of the Roman College, he bought a vineyard at the foot of the Aventine hill […]. He did this in a time of great economic difficulties. He built a house there or remodeled one already existent” (Dalmases, 1985, p. 259). These students were primarily young Jesuits, although it cannot be ruled out that external students of the college also rested in the purchased villa.

Both Ignatius’s manner of conduct and his orders and letters convey the unmistakable conviction that sick confreres, including professors and students, are to be treated in a privileged manner to enable them to regain the health necessary to fulfill the duties entrusted to them. This is one of the main tasks of the rector, as Ignatius writes in a letter to the Jesuits sent to Ingolstadt (June 9, 1556) to open a college there: “The superior (in this case the rector) will strive to ensure that all his people maintain health and physical strength, necessary to undertake the hardships of God’s service. Therefore, he cannot allow them to overwork themselves with studies, other duties, or spiritual exercises. They are to observe moderation in everything [*tutto sia moderato*], according to the capabilities of each person, place, and time” (Loyola, 1911, p. 368).

It was clear in Ignatius’s directives that sick Jesuits, whether they were academic staff or students, were to receive preferential treatment, even if it incurred additional costs and necessitated replacements at work. The superior of each facility was tasked with overseeing this. Furthermore, Ignatius, renowned for his pragmatism and organizational abilities, instituted distinct roles within the Jesuit community to guarantee adequate care for the sick. These positions included the prefect of health, the infirmarian (nurse), and the pharmacist. Their duties involved assisting the rector in overseeing the health requirements of the community, thereby ensuring the smooth and proficient care of ailing members.

## Responsibilities of the Health Prefect: The Representative of the Rector for Health Matters

### The Role and Origin of the Health Prefect

The prefect of health (*praefectus sanitatis* or *procurator sanitatis*), acting as the rector’s representative for health matters, played a crucial role in larger Jesuit institutions, particularly in colleges where professors and students resided. Their primary task was to ensure the well-being of all residents and promptly inform their superior about any health issues that arose. In the efficiently managed Jesuit Order, each position had its own defined scope of responsibilities, and the prefect of health was no exception.

The rules of operation for the prefect of health were established early on, with the oldest set dating back to 1556 and formulated in Italian with the Latin title Regulae procuratoris sanitatis (1948). Comprising 12 guidelines for the care of the sick, these rules were initially implemented at the *Collegium Romanum*. However, Ignatius extended the mandate of appointing a prefect of health to other colleges within the Jesuit Order. Consequently, all institutions adhered to the same set of rules, ensuring consistency in the care provided across Jesuit establishments.

### Core Duties and Weekly Responsibilities

The rules comprised a well-conceived and orderly set of guidelines for action. The primary duty of the prefect of health was to ensure the well-being of all residents of the college, including the *Scolari*, with the ultimate aim being “the greater glory of God.” The prefect was instructed to give special attention to those who were weaker or engaged in strenuous work (no. 1).

The prefect was required to inquire about the well-being of everyone and address any needs for clothing and footwear essential for maintaining health on a weekly basis (no. 2). Additionally, they were to liaise with the house buyer and those responsible for meal service to ensure that all brothers were eating adequately, without either excessive indulgence or undue restraint (no. 3–6). If any Jesuit required better food, clothing, or bedding due to health concerns or excessive exertion, the prefect was to inform the rector (no. 7).

Furthermore, the prefect was responsible for ensuring that Jesuits found time for physical exercise in the garden (no. 9) and had an hour of daily recreation. If the prefect deemed a Jesuit unfit to assist in the kitchen, they were to report this to the rector (no. 10). In the event of illness, it was the duty of the prefect to summon a doctor after consulting with the superior (no. 12).

The practice of calling a doctor for a sick person, which may seem obvious today, was not so during Ignatius’s time. In that era, there persisted a belief inherited from medieval orders that excessive care for the body posed a threat to the soul. For instance, Bernard of Clairvaux (1090–1153) explicitly instructed his fellow Cistercians not to seek medical assistance or take medications when feeling ill (Welie, 2003).

The message conveyed in this comprehensive Jesuit document is straightforward: maintaining good health is a fundamental, albeit instrumental, matter crucial for the effective execution of the order’s mission. Consequently, a specialized position is established within the college to ensure that everyone, tailored to their individual health condition and type of work, properly nourishes themselves, attends to their physical well-being, and obtains necessary rest.

### Refining the Role and Regulations of the Prefect of Health

The actions undertaken by the prefect of health serve as a commendable example of implementing the principle of *cura personalis*, which was formally articulated only in the twentieth century but has been ingrained in the order’s spirituality since its inception (Casalini, 2020; Marek & Walulik, 2022). The Society of Jesus attends to the entirety of a *person*, including their physical dimension, in a highly *person*alized and individualized manner.

The regulations pertaining to the prefect of health were published in abbreviated Latin form in subsequent centuries as Regulae praefecti sanitatis (1870). An important addition to these regulations was the inclusion of a rule stating that a nurse, known as an infirmarian, subordinate to the prefect, is tasked with directly caring for sick members of the household. Nonetheless, this role existed practically from the inception of the order’s activities.

## Rules for the Infirmarian: Nursing Guidelines for Sick Jesuits

### Qualifications and Attributes of the Infirmarian

The role of the infirmarian (nurse, *infirmarius*) was assigned to Jesuit brothers who possessed suitable physical attributes—being tall and strong—as well as positive mental qualities such as “cheerfulness and kindness” (Grzebień (Ed.), 1996, p. 229). They were also bound by detailed regulations, serving as a comprehensive guide to nursing practices of that time. These rules were formulated in the mid-sixteenth century and initially published in Spanish between 1553 and 1554 by Father Jérôme Nadal, a close collaborator of Ignatius of Loyola (Regulae infirmarii, 1948). Over subsequent centuries, these regulations underwent slight modifications, additions, and adaptations to suit local conditions. Nadal’s original version comprised three parts, each outlining specific responsibilities and protocols.

### Spiritual Care: The Primary Focus

The first part (pp. 441–444) commences with instructions for the infirmarians concerning their approach, initially focusing on the spiritual well-being of the sick person before attending to their physical needs. The nurse’s foremost responsibility is to uplift the patient’s spirit, urging them toward absolute obedience to the doctor (no. 4) and fostering an attitude of patience and humility (no. 1), which will serve as an example for visitors (no. 5). The infirmarian is tasked with reinforcing the patient’s faith in God, emphasizing that it is he who visits the sick through illness (no. 1). Additionally, the nurse should encourage the patient to engage in religious practices, including making confession, receiving Holy Communion, and, if necessary, receiving extreme unction (no. 2). In the event that the patient’s condition deteriorates, the infirmarian is obligated to inform their superior, who will then organize prayers for the sick individual within the religious community (no. 3).

The strong emphasis on caring for the patient’s spiritual dimension can be attributed to two main factors. First, it stems from Ignatius’s belief that all life events, including illness, are ordained by God and represent spiritual challenges, necessitating prayerful reflection. Second, it reflects the prevailing concept of humanity during that time, which differed from Cartesian dualism. This concept views the body (*res extensa*) and the human mind (*res cogitans*) as interconnected rather than independent and separate entities. The Jesuits, drawing from the Gospel (Matthew 9:1–8), held the conviction that human healing was holistic: addressing spiritual well-being preceded physical healing.

### Physical and Emotional Care Practices

The subsequent sections of the first part of the *vademecum* (pp. 442–444) detail instructions on how the infirmarian should attend to the physical and mental aspects of the individual afflicted by illness. First, the nurse must ensure that the patient receives all prescribed medications from the doctor without any alterations (no. 1). Similarly, the same principle applies to meal type and timing (no. 2), with an emphasis on providing tasty and delicate meals made from fresh ingredients (no. 3). The patient is to rest in a pedantically prepared bed (no. 3) within a meticulously clean room adorned with plants (*yerbas*) and other items that bring joy to the patient (no. 4). If deemed necessary by the doctor, the nurse should also tend to light the stove. Additionally, the nurse should engage the patient in cheerful conversation, entertain them with jokes, and read pious books to ensure that their time is spent fruitfully (no. 5).

At this juncture, a provision is made with soberness and firmness: the nurse is instructed to ascertain the patient’s preferences regarding visitors and admit only those patient desires (no. 5). Once the doctor permits the patient to leave their bed, the nurse continues monitoring their health and diet until the superior deems the risk of relapse minimal (no. 8).

### Oversight of Convalescents and At-Risk Residents

The second, shorter part of the rules (p. 444) outlines the approach of the infirmarian toward healthy individuals. Special attention should be given to convalescents and those weakened by age or previous illnesses. The nurse, in collaboration with the minister of the house, must ensure that they adhere to the dietary and activity restrictions prescribed by the doctor (no. 1). Individuals who have just completed long and strenuous Ignatian exercises receive similar treatment (no. 2). Overall, nurses are responsible for the well-being of everyone and are obligated to report any factors that may pose a risk of illness to their superior (no. 3).

### Professional Ethics and Practical Guidelines

The third and most extensive part of the rules, spanning 14 points (pp. 444–447), delineates the specific deontology for the nurse during that period. Foremost, the *infirmarius* should embody the qualities of love, discretion, and piety, demonstrating obedience to superiors and kindness toward others (no. 1). Endurance of the unpleasantness and inconveniences inherent in the profession is expected from the infirmarian, all while striving to alleviate the suffering of the sick (no. 2). The prioritization of personal health is emphasized, particularly when infirmarians are in contact with individuals afflicted by infectious diseases.

It is advised not to undertake tasks or care beyond one’s physical capabilities (no. 3). If necessary, assistance should be sought for meal preparation and other services for the sick (no. 4). Should any household member feel unwell, consulting the minister of the house and, if deemed necessary, summoning a doctor and preserving the patient’s urine for testing are imperative (no. 5).

During the doctor’s visits, the nurse should be present to better comprehend medical recommendations, particularly regarding medications and diet, and to document pertinent information (no. 6). Consultation with the minister of the house is recommended before taking any action, especially in areas of uncertainty (no. 7). Keeping the doctor informed about the onset and resolution of symptoms, as well as temperature fluctuations, facilitates decisions regarding meal provision (no. 8). Vigilance in ensuring that prescribed medicines are dispensed by the brother pharmacist and are of satisfactory quality is essential (no. 9).

Fluctuations in a sick person’s temperature should be reported to the superior (no. 10). In cases of severe illness, consultation with the minister of the house regarding the need for an additional doctor is advised (no. 11). Maintaining a barber acquaintance in the city for bloodletting, if needed, is recommended (no. 12). Regular reviews of these rules to prevent forgetfulness and documentation within the infirmary are encouraged (no. 14).

### Additional Roles: Pharmacy Management

The text includes four additional rules. The first addresses the spiritual disposition of the infirmarian, while the second assigns auxiliary tasks in the pharmacy if one exists at the college. The nurse is responsible for overseeing the distillation of water (no. 2) and meticulously cleaning all equipment, instruments, and jars monthly, following the pharmacist’s guidance (no. 3). Additionally, the nurse is tasked with maintaining a pharmacy book, documenting the contents of individual jars and the inventory.

Understanding the exact manner in which each infirmarian carried out his responsibilities presents a challenge. However, it is reasonable to assume that they strived to meet the expectations laid out in the rules as they reflected on their daily actions during the Examination of Conscience (Fuchs et al., 2023). In many cases, the roles of the infirmary attendant and pharmacist were merged and fulfilled by the same individual. This arrangement constituted a demanding full-time job that required significant dedication and effort.

### Infirmary Operations and Scope of Care

An important clarification is necessary when discussing the operation of infirmaries within Jesuit colleges. The infirmaries were typically situated behind the cloister and were designated solely for sick Jesuits. However, there are indications that this rule was frequently disregarded, especially in the case of certain lay students. In Polish territories, subsequent provincials reiterated the infirmary’s purpose exclusively for Jesuits in their post-visitation memoranda (e.g., Tomasz Smaga in the regulation for the Poznan college dated December 28, 1645) (*Memorialia Collegio Posnaniensi relicta*, 1645, f. 48), indicating instances of rule-breaking.

It is evident that the patient population of the infirmary comprised, among others, students residing in Jesuit music seminaries. This is because many infirmarians not only provided medical treatment but also served as prefects or assistants in these seminaries. Additionally, in some colleges, dormitories were supervised by pharmacists, who frequently offered student's assistance when they fell ill. Consequently, in practice, a sick resident of a boarding school would either seek treatment at the Jesuit infirmary or be visited in their room by an infirmarian or pharmacist.

In the case of student's residing in the *Collegium Germanicum* in Rome, historical records from 1564 indicate a specific protocol. If a student fell ill, the rector was instructed to promptly inform the parents and arrange the student’s return home. However, if the parents resided at a considerable distance, the tutor (referred to as the *gentiluomo*) of the sick student was tasked with finding a suitable place for recovery, covering expenses for medicine and a doctor. Notably, the rector was explicitly cautioned against offering to cover medical expenses, emphasizing that such costs should be borne by the parents or tutor of the sick student (Goisson, 1974).

However, less attention was given to students living in private dormitories and even less to those in family homes. Nonetheless, it is conceivable that the Jesuit medical staff also had an interest in them. This assumption is reinforced by the fact that if a student were absent from school, the lecturer would promptly dispatch one of their student assistants, known as a *censor*, to the student’s family home or private residence to inquire about their well-being (Schlederer, 2000).

### Broader Implications of Jesuit Medical Care

Therefore, it can be inferred that the regulations applicable to Jesuit medical staff also extended, to some extent, to their approach toward all sick students of the college. It is improbable that their illness would be disregarded by the Jesuits, who not only exerted considerable effort in their upbringing and education but also sought to maintain a favorable impression of themselves among the students’ parents. This was especially significant during the era of the Roman Catholic Church’s efforts to reclaim territories lost to Protestants.

## The Responsibilities of the Pharmacist

The team responsible for caring for sick Jesuits and students residing in boarding houses at Jesuit colleges consisted of three key officers—the prefect of health, the infirmarian, and the pharmacist—potentially assisted by others. While their primary focus was the well-being of the Jesuit community, they likely also attended to other students within the college. These individuals received comprehensive education and training, positioning them as an elite group among Jesuit brothers.

They had direct access to patients, including those in influential positions within the order or from higher social classes. Therefore, they were expected to exhibit professionalism characterized by patience, tact, and discretion. However, they understood their role as auxiliaries to professional doctors, whom they would call upon, in more severe cases (Padberg, 1996, no. 304).

As previously mentioned, it was common for the pharmacist to also serve as an infirmarian, leading to the absence of separate regulations specifically for pharmacists in Jesuit documents. However, five rules were initially established (Regulae coimbricenses, 1948) for the custodian of medicines (*medicamentorum custos*) at the Jesuit “college” established in 1542 in Coimbra, Portugal. This institution, although not a traditional college, served as a residence for Jesuits attending the University of Coimbra. Eventually, these rules were fully assimilated into the infirmary regulations developed by Nadal.

### Pharmacy Operations and Public Service

Pharmacies have been in operation at numerous former Jesuit colleges since the late sixteenth century. While infirmaries primarily catered to sick Jesuits, pharmacies served a broader clientele. This included students of the college, benefactors of the order, various dignitaries, and even impoverished inhabitants of the city who received medicines free of charge (Grzebień (Ed.), 1996). Jesuit brothers served as pharmacists in these establishments, and in larger pharmacies, they often received assistance from other brothers and occasionally lay individuals.

### Education and Professionalism of Jesuit Pharmacists

Managing a pharmacy was a lifelong commitment, unlike other roles within the order, as it demanded extensive knowledge and professional experience. Pharmacists, initially referred to by the Greek term *pharmacopola* and later by the Latin word *apothecarius* until the mid-seventeenth century, constituted the most educated group among Jesuit brothers (Mariani, 2021).

Obituary records indicate that some of these pharmacists also undertook the duties of a surgeon (Grzebień (Ed.), 1996), tasks typically not performed by individuals holding higher orders, such as priests. In fact, church law prohibited priests from practicing medicine or surgery, a restriction outlined as early as Canon 18 of the Fourth Lateran Council in 1215. Exceptions to this prohibition were only granted in special circumstances and in mission territories, subject to the special permission of the Pope (*sine apostolico indulto medicinam vel chirurgiam ne exerceant*: *The 1917 or Pio-Benedictine Code of Law*, 2001, Canon 139, § 2).

Jesuit pharmacists employed an eclectic approach to treatment, utilizing both traditional galenic formulations and iatrochemical preparations (Mariani, 2021). Their pharmacies were well-stocked and offered a wide array of medications. These included affordable medicines derived from domestic plants designed to aid the less affluent, as well as more exclusive preparations containing imported raw materials for the nobility and magnates.

An original list dating back to the mid-eighteenth century, containing 267 medicines from the Jesuit Pharmacy in Glatz, has been preserved. This pharmacy belonged to the Czech province of the Society of Jesus in 1754 before being transferred to the Silesian province. The document, written in Latin, categorizes the specifics into various sections such as “*Extracta*” (extracts), “*Olea Coctas et Destill*” (non-distilled and distilled oils), “*Praeparatae*” (preparations), “*Radices*” (roots), “*Salia*” (salts), and “*Semina*” (seeds) (Lec, 2014). The quantity of medicines and raw materials stocked in Jesuit pharmacies in the Grand Duchy of Lithuania was even greater, ranging from approximately 350 items in Slutsk to over 1,300 in Pinsk (Mariani, 2021).

### The Creation of Jesuit Remedies

Among the medications stocked in Jesuit pharmacies were remedies produced by the Jesuits themselves, crafted from wild herbs or cultivated in special gardens located at some Jesuit residences associated with colleges. One notable example in Poland was the creation of “Jesuit pills” in 1591 by Brother Wilhelm Lambert SJ, an English pharmacist. These pills were believed to be effective against “plague air,” and when six Jesuits in Krakow became infected in March 1592, five of them recovered after taking these pills (Zaleski, 1905, p. 295). Other well-known medicines include “miracle *vel* Jesuit medicine” or Jesuit powder (*Pulvis jesuiticus*) and Jesuit tea, which were used in Poland during the early twentieth century (Sobolewski, 2018). However, it is important not to confuse these with powdered cinchona bark (*pulvis corticis chinae de china vel peruvianus*), imported from Peru by the Jesuits. This substance quickly emerged as a reliable remedy against malaria (Knapp, 2002). It was introduced to Europe by the Jesuit Cardinal Juan de Lugo and the pharmacist Brother Piero Paulo Pucciarini during the 8th General Congregation of the Society of Jesus, held in Rome in 1645. Within 15 years, the Jesuits had disseminated it across the continent (Prinz, 1989). Cinchona bark is one of the few medicines that have been used effectively for several centuries. In 1820, quinine was extracted from cinchona bark, and it continues to be utilized today for the treatment of complicated cases of malaria (Prinz, 1989).

## Discussion

The present research clearly demonstrates that in former Jesuit colleges, the care provided to sick teachers and students was distinctive. This care, deeply rooted in the Christian religion, especially the spiritual teachings of Ignatius of Loyola, was formalized in regulations for Jesuit medical personnel. Analyzing these rules not only sheds light on the approach to incapacitated members of the academic community at the time but also reveals surprising parallels with later centuries’ approaches based on scientific advancements.

### The Healing Atmosphere of Religious Settings

The process of tending to sick residents of Jesuit colleges, including both teachers and students, unfolded within a religious atmosphere. The medical staff approached their duties with a religious mindset and aimed to instill the same attitude among the sick. Integral to this approach was the interpretation of illness as a divine gift, offering both an opportunity for spiritual transformation and a challenge to be vigorously combated to regain health. This dual perspective emphasized the potential for individuals to return to health and thereby serve society and the Church more effectively. The religious environment surrounding the care of the sick contributed to their potential for swifter recovery.

Currently, many researchers recognize the positive influence of religiosity on the healing process (Koenig et al., 2024). They assert that religious beliefs and practices may influence one’s ability to cope with stress, the risk of depression, and levels of anxiety or fear. Religious rituals or rites (e.g., sacraments) and religious interventions may similarly reduce negative emotions and enhance positive emotions, thereby impacting physical health. However, the usefulness of a religious climate for recovery does not imply that medical personnel have the right to introduce religion into people’s lives or attempt to convert them.

### Early Insights into Holistic Nursing Practices

In their approach to caring for sick college residents, the Jesuits placed a strong emphasis on maintaining cleanliness, providing nutritious food, and ensuring a supportive environment, recognizing the importance of these factors in promoting recovery and overall health. Similarly, in her seminal work *Notes on nursing* (Nightingale, 1860), Florence Nightingale emphasized the critical role of cleanliness, good nutrition, and a conducive environment for patient care. Nightingale advocated for meticulous hygiene practices to prevent infection, the importance of fresh and appealing food tailored to individual needs, and maintaining proper light and temperature to create a healing environment. Her insights highlight the enduring relevance of these principles, demonstrating their applicability beyond the Jesuit context and underscoring their foundational role in modern nursing practices.

### Prioritizing Mental Well-Being in Care

The staff responsible for caring for sick residents in colleges was instructed to prioritize the mental well-being of the sick individuals. This included decorating their rooms with plants, providing them with appetizing meals, engaging in pleasant reading and conversation, entertaining them with jokes, and permitting only positive company to visit. The aim of these measures was to expedite the recovery process.

Recent research corroborates the effectiveness of these intuitively applied methods, showing that maintaining good mental health—characterized by regular experiences of positive emotions and the development of healthy psychological traits—can significantly enhance physical health (Park et al., 2014; Trudel-Fitzgerald et al., 2019). Conversely, poor mental health, marked by negative emotions, emotional disorders, and psychological distress, has been shown to have the opposite effect (Scott et al., 2016). Psychological factors consistently impact physical health by influencing various bodily systems, including the central nervous system, autonomic nervous system, and endocrine system (specifically affecting cortisol, norepinephrine, and epinephrine stress hormone levels), as well as the cardiovascular and immune systems (Koenig et al., 2024).

### The Spiritual and Ethical Role of Caregivers

Former officers of Jesuit colleges tasked with caring for the sick were expected to embody qualities such as love, discretion, and piety, showing kindness and patience in their duties. Their motivation was to stem primarily from their spirituality. Recent research indicates that adherence to spiritual values continues to have positive effects on various aspects of employees’ behavior and well-being. These effects include increased altruism, consistency, reduced inter-role conflict, decreased frustration, higher organization-based self-esteem, greater involvement, and more ethical behavior (Maidl et al., 2022).

### Jesuit Colleges as Early Community-Based Care Models

Thanks to the care provided by the Jesuits for sick residents and students of the colleges, these individuals did not have to occupy beds in hospitals and poorhouses, which were primarily maintained by other orders (though sometimes by the Jesuits themselves), cities, or wealthy magnates. Care for the incapacitated in the colleges was, therefore, a form of community-based caregiving or in-home care, which—similar to today—saved considerable costs for healthcare services (Johnson et al., 2024). Given demographic changes, the pool of informal and formal caregivers is diminishing, and examining historical care models may assist public health in developing new solutions.

## Limitations

It is crucial to recognize the limitations in portraying the Jesuits’ approach to caring for sick members of former colleges. This article has primarily focused on colleges where academic staff and students resided. However, it must be acknowledged that the procedures outlined were generally applicable to all larger Jesuit institutions, even though most of them were colleges. Additionally, we relied heavily on existing regulations when presenting the protocols for dealing with sick teachers and college students. A more comprehensive investigation into how these regulations were put into practice and the actual provision of patient care would be beneficial. Despite this, considering the historical consistency of Jesuits in adhering to regulations, it is probable that the attitudes and actions of the Jesuit medical staff closely mirrored the prescribed rules.

## Conclusion

By understanding the historical context and the foundational practices of care in Jesuit colleges, we can appreciate the continuity and changes in these practices over time. This historical analysis has provided a comprehensive view that can inform contemporary discussions on healthcare in educational institutions. The study revealed that the approach to sick teachers and students in former Jesuit colleges was firmly grounded in the principles of the Christian faith, as updated by Ignatius of Loyola in his writings and normative documents. Understanding this aspect could shed light on the history of care for the sick and unveil its reliance on religious convictions.

This research has merely scratched the surface of these issues; further analysis could involve delving into additional Jesuit-related materials, such as handwritten histories of individual colleges and former students’ diaries (although, unfortunately, few have survived), to explore how care for the sick was practically implemented. Additionally, it would be intriguing to investigate how other schools and universities—both Catholic and Protestant—cared for sick teachers and students during the early modern period.
